# The endometrial transcriptome transition preceding receptivity to embryo implantation in mice

**DOI:** 10.1186/s12864-023-09698-3

**Published:** 2023-10-04

**Authors:** Hon Yeung Chan, Ha M. Tran, James Breen, John E. Schjenken, Sarah A. Robertson

**Affiliations:** 1 The Robinson Research Institute, School of Biomedicine, University of Adelaide, Adelaide, SA 5000 Australia; 2 Hunter Medical Research Institute, Infertility and Reproduction Research Program, New Lambton Heights, NSW 2305 Australia; 3 Priority Research Centre for Reproductive Science, School of Environmental and Life Sciences, Discipline of Biological Sciences, The University of Newcastle, University Drive, Callaghan, NSW 2308 Australia

**Keywords:** RNA sequencing, Uterus, Embryo implantation, Endometrial receptivity, Mouse

## Abstract

**Background:**

Receptivity of the uterus is essential for embryo implantation and progression of mammalian pregnancy. Acquisition of receptivity involves major molecular and cellular changes in the endometrial lining of the uterus from a non-receptive state at ovulation, to a receptive state several days later. The precise molecular mechanisms underlying this transition and their upstream regulators remain to be fully characterized. Here, we aimed to generate a comprehensive profile of the endometrial transcriptome in the peri-ovulatory and peri-implantation states, to define the genes and gene pathways that are different between these states, and to identify new candidate upstream regulators of this transition, in the mouse.

**Results:**

High throughput RNA-sequencing was utilized to identify genes and pathways expressed in the endometrium of female C57Bl/6 mice at estrus and on day 3.5 post-coitum (pc) after mating with BALB/c males (n = 3–4 biological replicates). Compared to the endometrium at estrus, 388 genes were considered differentially expressed in the endometrium on day 3.5 post-coitum. The transcriptional changes indicated substantial modulation of uterine immune and vascular systems during the pre-implantation phase, with the functional terms Angiogenesis, Chemotaxis, and Lymphangiogenesis predominating. Ingenuity Pathway Analysis software predicted the activation of several upstream regulators previously shown to be involved in the transition to receptivity including various cytokines, ovarian steroid hormones, prostaglandin E2, and vascular endothelial growth factor A. Our analysis also revealed four candidate upstream regulators that have not previously been implicated in the acquisition of uterine receptivity, with growth differentiation factor 2, lysine acetyltransferase 6 A, and N-6 adenine-specific DNA methyltransferase 1 predicted to be activated, and peptidylprolyl isomerase F predicted to be inhibited.

**Conclusions:**

This study confirms that the transcriptome of a receptive uterus is vastly different to the non-receptive uterus and identifies several genes, regulatory pathways, and upstream drivers not previously associated with implantation. The findings will inform further research to investigate the molecular mechanisms of uterine receptivity.

**Supplementary Information:**

The online version contains supplementary material available at 10.1186/s12864-023-09698-3.

## Background

As well as a developmentally competent blastocyst, implantation success depends on sufficient receptivity and responsiveness in the endometrial lining of the uterus [1, 2]. Receptivity is a transient state that develops following ovulation when the endometrium undergoes major cellular changes that impart receptivity to embryo adhesion, attachment and trophoblast invasion, enabling robust placental development to sustain fetal growth [3]. Although there are differences between mammalian species depending on reproductive strategy and anatomical differences, many features are broadly consistent. Acquisition of receptivity is primarily controlled by ovarian steroid hormones, notably elevated progesterone which drives a specific transcriptional program in endometrial epithelial cells and stromal fibroblasts to promote attachment competence and precede decidual transformation. Several critical molecular mediators of uterine receptivity have been described. Pro-implantation molecules, including vascular endothelial growth factor (VEGF), prostaglandin-endoperoxide synthase-2 (PTGS2), and leukaemia inhibitory factor (LIF), have been identified to play indispensable roles in embryo attachment, decidualization and uterine angiogenesis [4–6]. These agents are expressed under tight spatial and temporal regulation by ovarian hormones and have conserved roles in implantation in species including humans, hamsters, rats and mice [7–10].

Substantial changes in endometrial immune cells accompany transition to receptivity. This involves waves of recruitment of neutrophils, macrophages, and dendritic cells (DCs), followed by uterine natural killer (uNK) cells and regulatory T (Treg) cells [11–13]. These immune cells exert critical roles in mediating the decidual response, promoting vascular changes required to support placentation, suppressing inflammation, and mediating immune tolerance of fetal and placental alloantigens. In mice, it has been shown that seminal fluid factors delivered at mating contribute, along with ovarian steroid hormones, to regulating the immune changes that precede implantation [12, 14–19]. Treg cells are essential for maternal immune tolerance of the embryo allowing implantation to proceed [20]. Macrophages and DCs are also critical, as evidenced by defective implantation when these cells are depleted [21, 22]. The effects of macrophages and DCs are by virtue of their secretion of a wide range of tissue-remodelling and angiogenic factors, including VEGF, matrix metalloproteinases (MMPs), and tissue inhibitors of metalloproteinases (TIMPs), that facilitate uterine receptivity and embryo implantation [23, 24].

The mouse is commonly used as an experimental model to study regulation of implantation. Despite studies describing several key processes and factors involved in embryo implantation in mice, a comprehensive understanding of the transcriptional changes that occur in the uterus in order to acquire receptivity to embryo implantation, and the upstream signals that drive these changes, is lacking. Previous studies have focused on comparisons of uterine tissue compartments, for example comparing implantation sites and inter-implantation sites [25] at time points day 3.5 post-coitum (pc) (pre-implantation), day 4.5 pc (the time of implantation) and day 5.5 or 6.5 pc (post-implantation) [26–29], using microarray gene expression analysis or quantitative polymerase chain reactions (qPCRs) [29, 30]. While these comparisons focus on the molecular changes at or around the time of implantation, they do not provide information on the overarching molecular mechanisms by which the peri-implantation state evolves from its earlier peri-ovulatory state. Furthermore, microarray and qPCR technologies do not provide a complete transcriptomic profile of the uterus, as the techniques only detect genes using known RNA probes or primers, and do not detect novel transcripts.

The aim of this study was to generate a comprehensive profile of the endometrial transcriptome in the peri-ovulatory and peri-implantation states, in order to define a comprehensive assembly of the genes and gene pathways that are different between these states, and to identify new candidate upstream regulators of this transition, including any novel factors that might be delivered or otherwise affected by components of male partner seminal fluid. To obtain a complete transcriptome profile of the uterus before and after acquisition of receptivity, we utilized RNA-sequencing (RNA-seq) to compare transcriptomic profiles of the endometrium in mated female mice on day 3.5 pc to unmated estrous females. Here, we demonstrate that the pre-implantation endometrium shows a distinct gene expression pattern compared to the endometrium of estrous mice. Bioinformatics analysis indicates many cytokine and growth factor signaling networks are activated and predicts these to be associated with increased immune and angiogenic functions. Our data confirm that the non-receptive uterus undergoes major molecular changes before implantation to acquire receptivity and implicates several upstream drivers including ovarian steroid hormones and factors present in male seminal fluid. Amongst these are several factors not previously reported to be involved in the transition to endometrial receptivity. The results will inform future studies to unravel the underlying regulatory events and modifying factors.

## Results

### Differentially expressed genes in the endometrium at day 3.5 pc compared to estrus

**Fig. 1:**
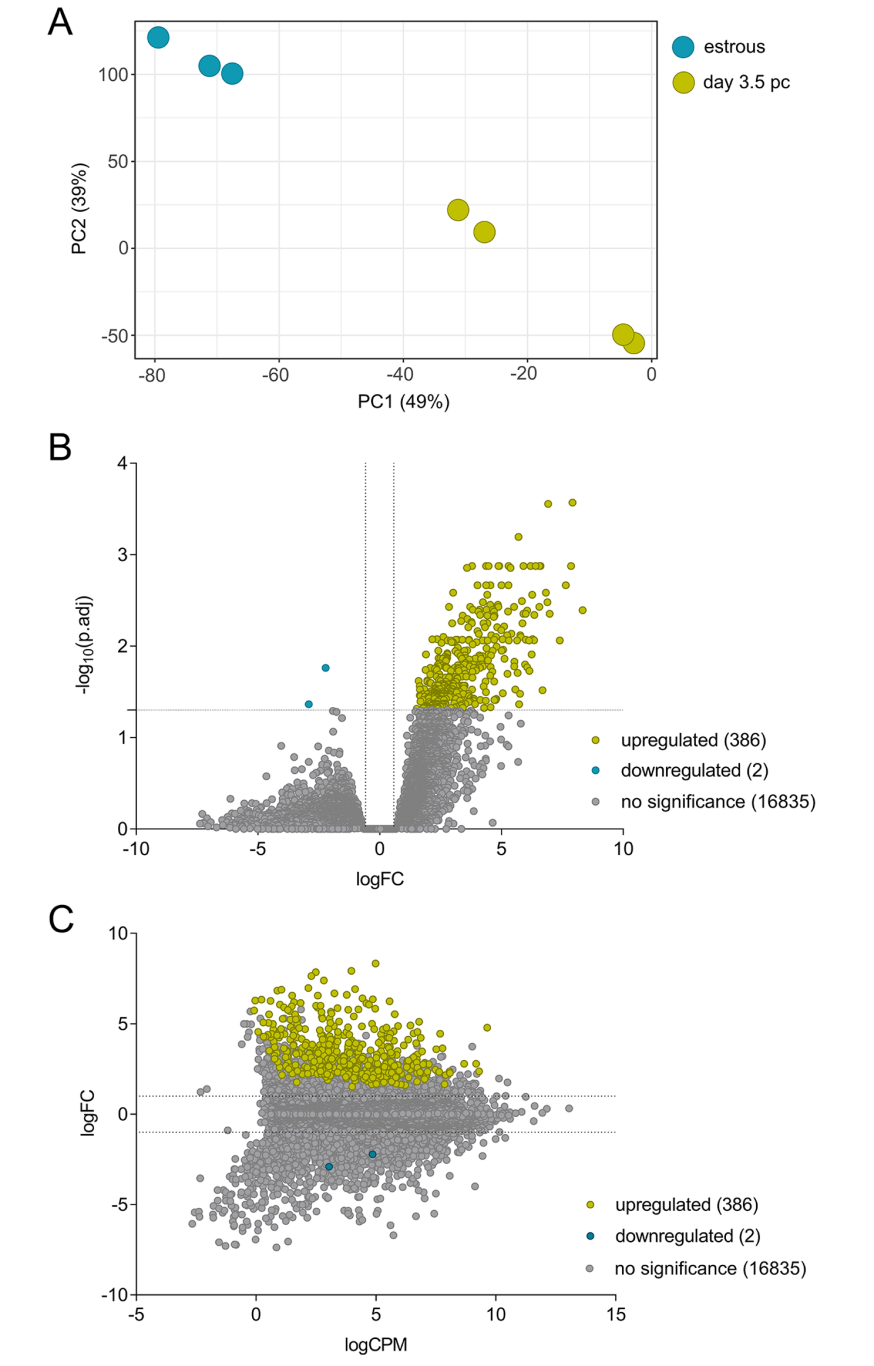
Transcriptomic changes in the day 3.5 pc endometrium compared to the estrous endometrium. Differentially regulated genes in the endometrium were identified using *limma/voom* and *edgeR* with a cutoff of FDR ≤ 0.05 and logFC ≥ +0.583 or ≤ -0.583 (equivalent of FC ≥ +1.5 or ≤ -1.5). **(A)** Principal component analysis of RNA-seq results visualizing the gene expression pattern of individual samples. **(B)** Volcano plot comparing logFC and -log_10_(FDR), and **(C)** MA plot comparing logFC and logCPM were plotted to identify specific genes that fit the threshold criteria. n = 3–4 samples/group

To evaluate changes in the endometrial transcriptome associated with acquisition of uterine receptivity, endometrial tissue was collected from C57Bl/6 female mice at estrus (peri-ovulatory phase) and on day 3.5 pc, around 12 h prior to embryo attachment and initiation of implantation (‘receptive’ phase). Tissues were subjected to RNA-seq analysis (n = 3–4 pooled samples per group: see Supplementary Material 1, Table S1 for RNA-seq metrics and Supplementary Material 1, Table S2 for expression of all transcripts detected). A total of 17,222 transcripts were detected after duplicates and lowly expressed transcripts (those with less than 3 counts per million mapped reads (CPM) detected in at least 3 samples per group) were removed. Following this, a principal component analysis (PCA) plot showed the data separated into two clusters corresponding to estrous and day 3.5 pc endometrium (Fig. 1A). R packages *limma-voom* and *edgeR* were used to identify differentially expressed genes using the criteria of log_2_ fold-change (logFC) ≥ +0.583 or ≤ -0.583 (equivalent of fold-change (FC) ≥ +1.5 or ≤ -1.5) and false discovery rate-adjusted p-value (FDR) ≤ 0.05, with 386 upregulated and 2 downregulated genes identified in the receptive endometrium compared to estrous endometrium (Fig. 1B). The average expression of these differentially regulated genes varied as shown by their log_2_CPM (logCPM), with many ranked in the mid- to low expression range and none ranked as highly expressed genes in the day 3.5 pc endometrium (Fig. 1C).

**Fig. 2:**
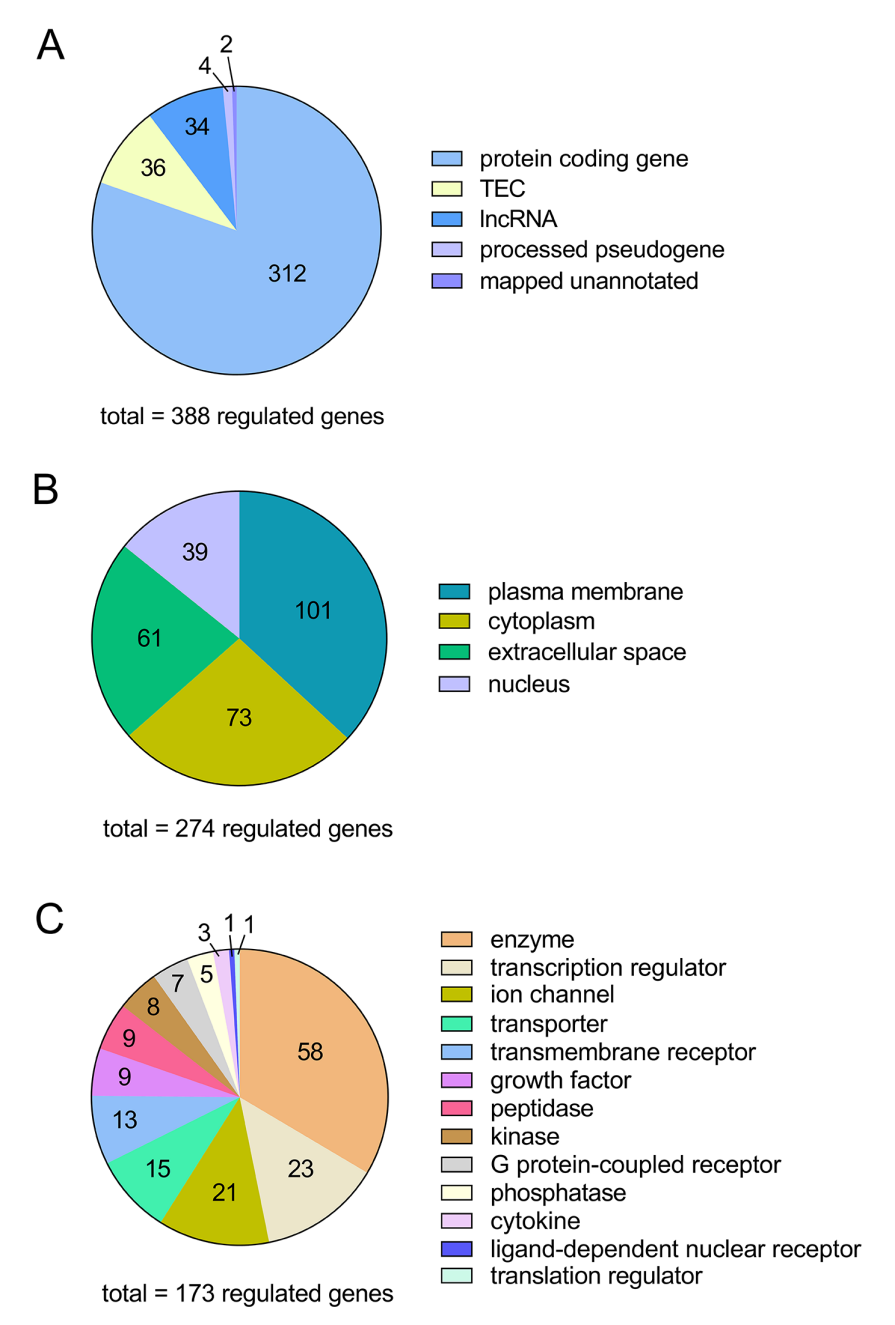
Characterization and localization of the differentially regulated genes in the day 3.5 pc endometrium compared to the estrous endometrium. **(A)** Molecule types of all differentially expressed genes identified by *limma-voom* and *edgeR*. IPA was used to characterize the **(B)** cellular localization of these molecules and **(C)** molecule types in the database. FDR ≤ 0.05 and logFC ≥ +0.583 or ≤ -0.583 (equivalent of FC ≥ +1.5 or ≤ -1.5). TEC, to be experimentally confirmed

Amongst the 388 differentially expressed transcripts were 312 protein-coding genes (80.4%), 36 predicted genes to be experimentally confirmed (TEC) (9.3%), 34 long non-coding RNAs (lncRNAs) (8.8%), 4 processed pseudogenes (1.0%), and 2 mapped unannotated genes (0.5%) (Fig. 2A). The top 10 upregulated genes and all downregulated genes, sorted according to logFC, are shown in Table 1. Notably, 1 of the top 10 upregulated genes is a predicted gene with as yet uncharacterised functions (Table 1). A complete list of all differentially expressed transcripts with gene name, description, biotype, accession number, logFC, and FDR value is provided in Supplementary Material 1, Table S2.

**Table 1:** Top 10 upregulated and all downregulated genes in the endometrium at day 3.5 pc (d3.5pc) relative to the endometrium at estrus (est)

Gene	Gene name	logFC (d3.5pc/est)	FDR
**Upregulated**			
*Acod1*	Aconitate decarboxylase 1	+8.33	0.004
*Gm32592*	Predicted gene, 32592	+7.92	<0.001
*Mdfic2*	MyoD family inhibitor domain containing 2	+7.85	0.001
*Cyp3a59*	Cytochrome P450, family 3, subfamily a, polypeptide 59	+7.64	0.002
*Adarb2*	Adenosine deaminase, RNA-specific, B2	+7.40	0.009
*St6gal2*	Beta galactoside alpha 2,6 sialyltransferase 2	+6.98	0.004
*Cxcl15*	Chemokine (C-X-C motif) ligand 15	+6.92	<0.001
*Ttr*	Transthyretin	+6.89	0.003
*Epha8*	Eph receptor A8	+6.83	0.003
*Ano2*	Anoctamin 2	+6.68	0.030
**Downregulated**			
*Adam33*	A disintegrin and metallopeptidase domain 33	−2.91	0.043
*Aspg*	Asparaginase	−2.22	0.017

To understand the localization and potential functions of the differentially expressed transcripts identified in the day 3.5 pc endometrium, Ingenuity Pathway Analysis software (IPA) was utilized and this enabled annotation of 384 of the 388 (99.0%) transcripts. IPA predicted these annotated transcripts to localize to the plasma membrane (101 genes, 26.3%), cytoplasm (73 genes, 19.0%), extracellular space (61 genes, 15.9%), and nucleus (39 genes, 10.2%) (Fig. 2B), while 110 genes (28.6%) were not assigned a location category (data not shown). Categorization of the differentially expressed transcripts identified 58 as enzymes (15.1%), 23 as transcription regulators (6.0%), 21 as ion channels (5.5%), 15 as transporters (3.9%), 13 as transmembrane receptors (3.4%), 9 as growth factors (2.3%), 9 as peptidases (2.3%), 8 as kinases (2.1%), 7 as G protein-coupled receptors (1.8%), 5 as phosphatases (1.3%), 3 as cytokines (0.8%), 1 as a ligand-dependent nuclear receptor (0.3%) and 1 translation regulator (0.3%) (Fig. 2C), while 211 genes (54.9%) were not assigned a specific functional category (data not shown).

### Canonical pathways regulated in the endometrium on day 3.5 pc compared to estrus

**Fig. 3:**
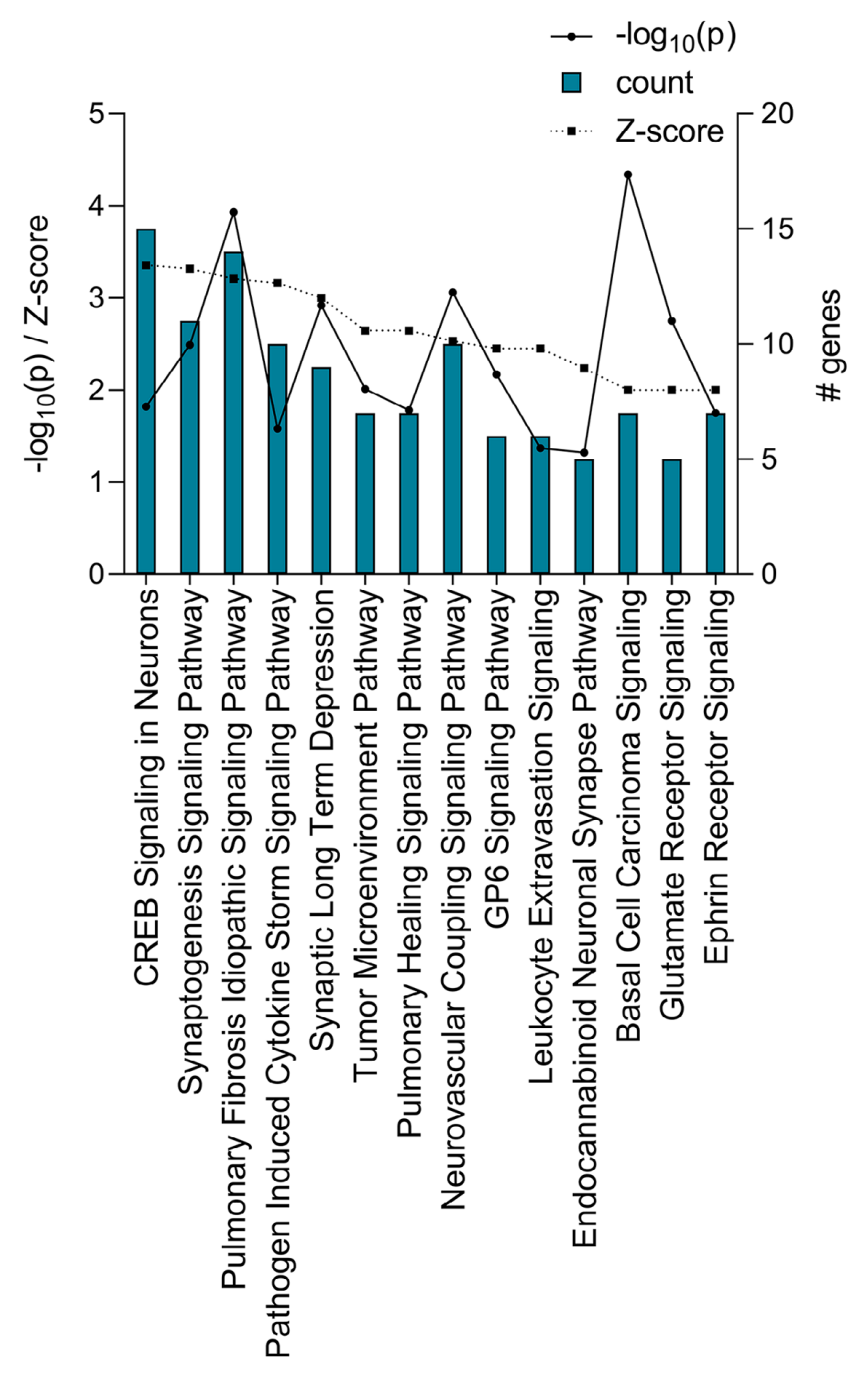
All regulated canonical pathways in the endometrium during the acquisition of receptivity identified by IPA. Canonical pathways predicted to be regulated in the endometrium on day 3.5 pc are shown, with -log_10_(p) and absolute value of Z-score on the left y-axis, and the number of differentially expressed genes that overlap with the corresponding pathway on the right y-axis. The 14 pathways identified were all predicted to be activated. All pathways are p ≤ 0.05 (equivalent of-log_10_(p) of +1.3) and Z-score ≥ +2.

To understand the functional significance of the differentially expressed genes identified in the day 3.5 pc endometrium, IPA was used to predict canonical pathways regulated by these genes. Using the cut-off criteria of p ≤ 0.05 and Z-score ≥ +2 or ≤ -2, 14 pathways were predicted to be activated in the day 3.5 pc endometrium compared to the endometrium at estrus, while no pathway was predicted to be inhibited (Fig. 3). Several immune regulatory pathways were amongst those identified as activated in the day 3.5 endometrium, including Pathogen Induced Cytokine Storm Signaling Pathway (10 genes, Z-score = +3.2), Tumour Microenvironment (7 genes, Z-score = + 2.6), and Leukocyte Extravasation Signaling (6 genes, Z-score = +2.4) (Fig. 3). In addition, pathways relevant to tissue and vascular remodeling such as Endocannabinoid Neuronal Synapse Pathway (5 genes, Z-score = +2.2), Pulmonary Healing Signaling (7 genes, Z-score = +2.6), and Neurovascular Coupling Signaling (10 genes, Z-score = +2.5) were predicted to be activated (Fig. 3). Differentially expressed genes that overlap with the identified activated pathways are shown in Supplementary Material 1, Table S3.

An alternative functional enrichment analysis using public domain analytical tools including Gene Ontology (GO), Kyoto Encyclopedia of Genes and Genomes (KEGG), and Reactome revealed enrichment of biological terms associated with known cellular changes in the uterus prior to implantation (the top 15 terms are provided in Supplementary Material 2–4, Figure S1–S3, and a full list of terms with aligned genes are listed in Supplementary Material 1, Table S4–S6). These enriched biological processes and molecular functions included Cell-cell adhesion via plasma-membrane adhesion molecules, Epithelial cell proliferation, Cell adhesion molecule binding, Hedgehog signaling pathway and Focal adhesion (Supplementary Material 2 and 3, Figure S1 and S2). Terms related to the extracellular matrix (ECM) such as ECM organization, ECM-receptor interaction, and Degradation of the ECM, and cellular component Collagen-containing ECM, were enriched in the day 3.5 pc endometrium (Supplementary Material 2 and 4, Figure S1 and S3).

### Upstream molecules regulated in the endometrium on day 3.5 pc compared to estrus

**Fig. 4:**
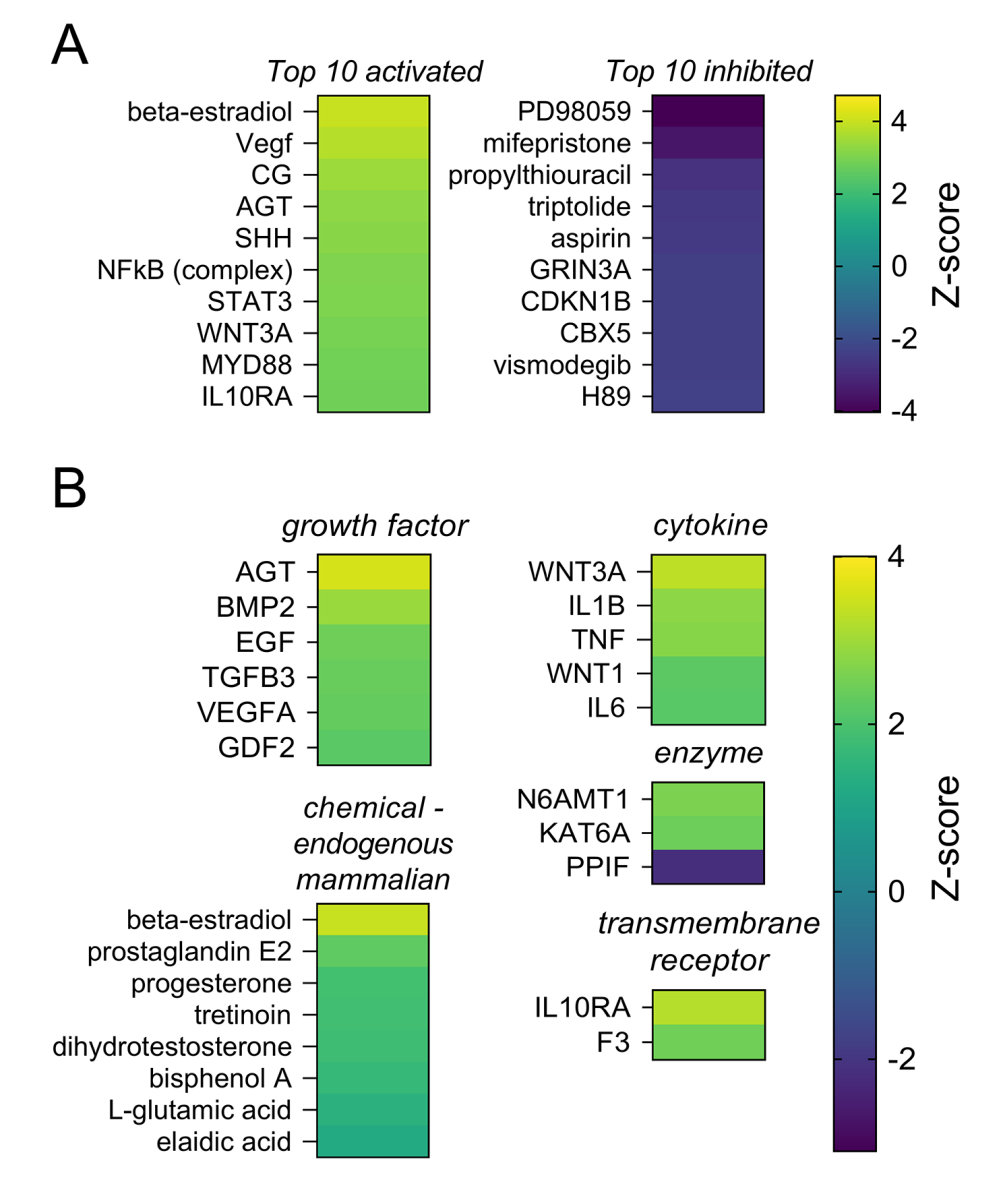
Molecules predicted to be upstream regulators of transcripts differentially expressed in the endometrium during the acquisition of receptivity, identified by IPA. **(A)** Heatmaps of the top 10 activated and top 10 inhibited upstream regulators. **(B)** Heatmaps were generated for the categories of cytokine, enzyme, growth factor, transmembrane receptor, and chemical – endogenous mammalian. p ≤ 0.05 and Z-score ≥ +2 or ≤ -2 for all molecules

To predict upstream regulators implicated in contributing to acquisition of endometrial receptivity, upstream regulator analysis in IPA was carried out. Using the cut-off criteria of p ≤ 0.05 and Z-score ≥ +2 or ≤ -2, 107 molecules were predicted to be upstream drivers of the gene expression changes in the endometrium on day 3.5 pc compared to estrous endometrium (Supplementary Material 1, Table S7), with the top 10 activated and top 10 inhibited upstream regulators shown in Fig. 4A. Amongst the top 10 activated molecules were beta-estradiol, vascular endothelial growth factor A (VEGFA), angiotensinogen (AGT), and chorionic gonadotrophin (CG). In particular, activation of the toll-like receptor 4 (TLR4) pathway was evident, including the target nuclear factor-κB (NFκB) and the adaptor protein myeloid differentiation primary response 88 (MYD88) (Fig. 4A). The chemical drug mifepristone, a progesterone antagonist, was notable amongst the top 10 inhibited molecules (Fig. 4A), consistent with the known role of progesterone signaling in the acquisition of uterine receptivity [31].

To investigate the upstream regulators likely to be most relevant to reproductive function, we focused on molecules in the categories of endogenous mammalian chemical, cytokine, enzyme, growth factor, and transmembrane receptor and sorted these by Z-score (Fig. 4B). A total of 24 upstream regulators were identified (23 predicted to be activated and 1 predicted to be inhibited, Supplementary Material 1, Table S7). Signaling pathways for the ovarian sex hormones known to be essential for regulation of uterine receptivity, beta-estradiol and progesterone [32–34], were predicted to be activated upstream of the differentially regulated genes (Z-score = +4.1 and +2.6 respectively). Interestingly, signaling by the androgenic sex hormone dihydrotestosterone was also predicted to be activated (Z-score = +2.6) (Fig. 4B). Signaling by growth factors associated with angiogenesis and vascular functions, including epidermal growth factor (EGF) and VEGFA were predicted to be activated [35], with Z-scores of +2.4 and +2.3 respectively (Fig. 4B). Furthermore, growth factors including prostaglandin E2 (PGE2), bone morphogenetic protein 2 (BMP2), and transforming growth factor beta 3 (TGFB3), all previously implicated in embryo implantation, the decidual response, and immune functions respectively [36–38], were predicted to be activated with Z-scores of +3.0, +2.9 and +2.4 (Fig. 4B). Pro-inflammatory cytokines interleukin (IL)-1B, IL6, and tumor necrosis factor (TNF) were also predicted to be activated (Z-score = +2.8, +2.2, +2.7 respectively) (Fig. 4B), consistent with substantial evidence that a pro-inflammatory state contributes to endometrial receptivity to embryo implantation [39, 40].

To identify molecules predicted to be regulators in the early receptive endometrium that have not been previously reported or well investigated in uterine receptivity, a PubMed search was conducted using the name of molecules identified as upstream regulators in Fig. 4B and ‘uterine receptivity or embryo implantation’ as search terms (e.g. WNT3A AND (uterine receptivity OR embryo implantation)) and the number of search results was recorded (Supplementary Material 5, Figure S4). There were 17 molecules that returned with > 1 search result, with progesterone having the highest number of search results, followed by beta-estradiol, EGF, IL6, and TNF (Supplementary Material 5, Figure S4). There were 3 molecules that returned only 1 search result, including elaidic acid, IL10 receptor subunit alpha (IL10RA), and AGT (Supplementary Material 5, Figure S4). Another 4 molecules returned no search result, including growth differentiation factor 2 (GDF2), peptidylprolyl isomerase F (PPIF), lysine acetyltransferase 6 A (KAT6A), and N-6 adenine-specific DNA methyltransferase 1 (N6AMT1), suggesting these agents may have novel regulatory roles in endometrial receptivity (Supplementary Material 5, Figure S4).

### Physiological functions regulated in the endometrium at day 3.5 pc compared to estrus

**Fig. 5:**
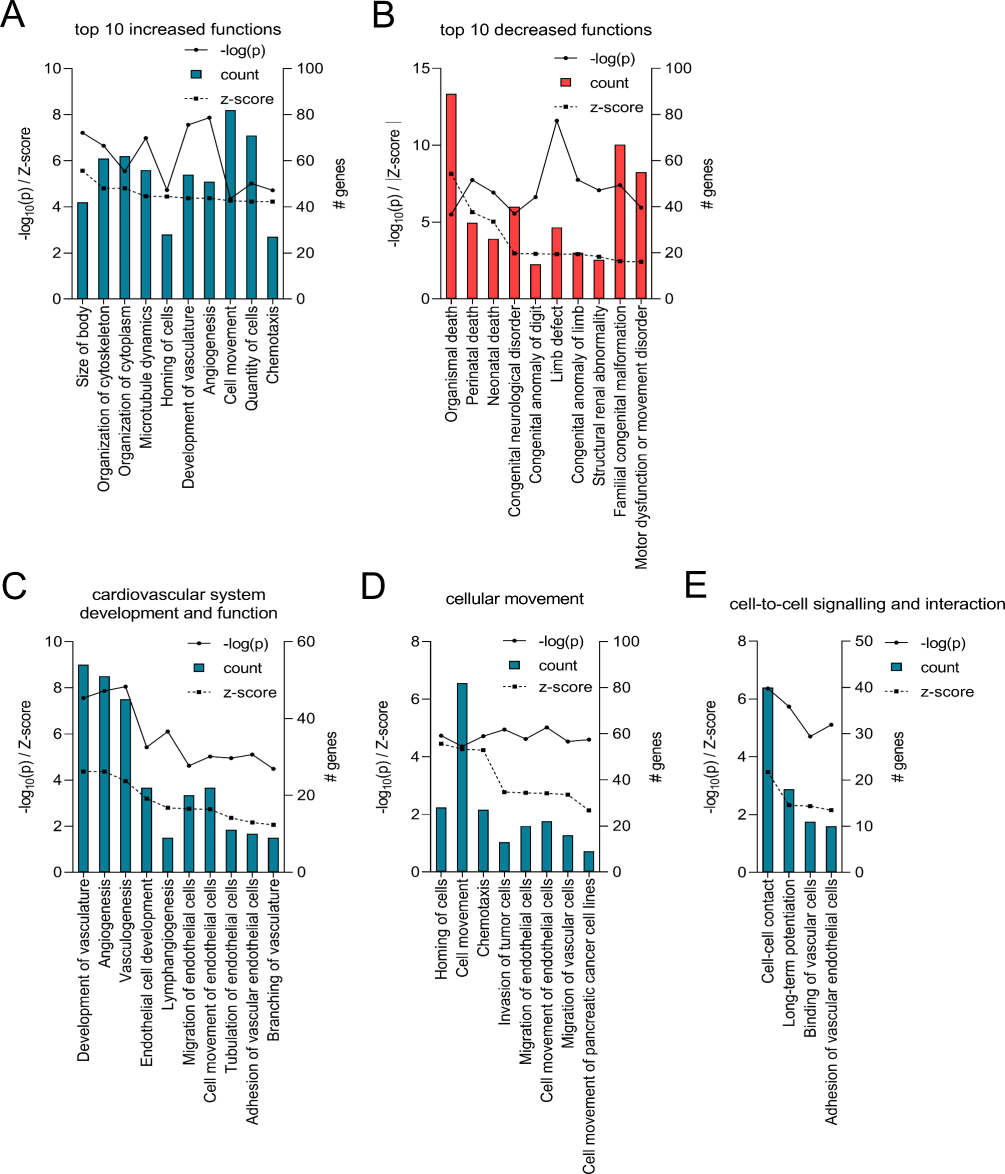
Biological and disease functions regulated in the endometrium during the acquisition of receptivity identified by IPA. **(A)** Top 10 increased and **(B)** top 10 decreased biological and disease functions to be regulated based on the differentially regulated molecules in the endometrium on day 3.5 pc. To focus on functional terms related to embryo implantation, all terms in **(C)** Cardiovascular system development and function, **(D)** Cellular movement and **(E)** Cell-to-cell signaling and interaction are listed. -log_10_(p) and absolute value of Z-score are shown on the left y-axis, and the number of differentially expressed genes that overlap with the corresponding function is shown on the right y-axis. Increased functional terms are shown as blue bar and decreased terms are shown as red bars. p ≤ 0.05 (equivalent of -log_10_(p) of 1.3) and Z-score ≥ +2 or ≤ -2 for all functions

The physiological functions regulated by the differentially expressed genes were predicted using the ‘Functions and Diseases’ analysis in IPA. There were 89 terms that met the cut-off of p ≤ 0.05 and Z-score ≥ +2 or ≤ -2, with 75 functions predicted to be increased and 14 predicted to be decreased (Supplementary Material 1, Table S8). Amongst the top 10 increased functions (Fig. 5A), terms related to vascular functions including Development of vasculature (Z-score = +4.4) and Angiogenesis (Z-score = +4.4) are enriched, consistent with the vascular changes known to precede and accompany implantation. On the other hand, Organismal death (Z-score = -8.1), Perinatal death (Z-score = -5.6) and terms associated with developmental disorders, including Congenital anomaly of digit (Z-score = -2.9), Limb defect (Z-score = -2.9), Congenital anomaly of limb (Z-score = -2.9) and Structural renal abnormality (Z-score = -2.7), are amongst the top 10 decreased terms (Fig. 5B).

To focus on functions related to vascular and immune response that are essential to the acquisition of uterine receptivity, all terms in the following 3 categories were selected: (1) Cardiovascular system development and function, (2) Cellular movement and (3) Cell-to-cell signaling and interaction (Fig. 5C-E). Indeed, in addition to the afore-mentioned top 10 increased functions, other vascular functions including Vasculogenesis (Z-score = +4.0), Migration of vascular cells (Z-score = +2.7), Binding of vascular cells (Z-score = +2.3), Adhesion of vascular endothelial cells (Z-score = +2.2) and Branching of vasculature (Z-score = +2.1), were predicted to be increased in the endometrium at day 3.5 pc compared to estrus (Fig. 5C-E). Similarly, immune functions including Chemotaxis (Z-score = +4.3) and Lymphangiogenesis (Z-score = +2.8) were predicted to be increased (Fig. 5C-D).

## Discussion

**Fig. 6:**
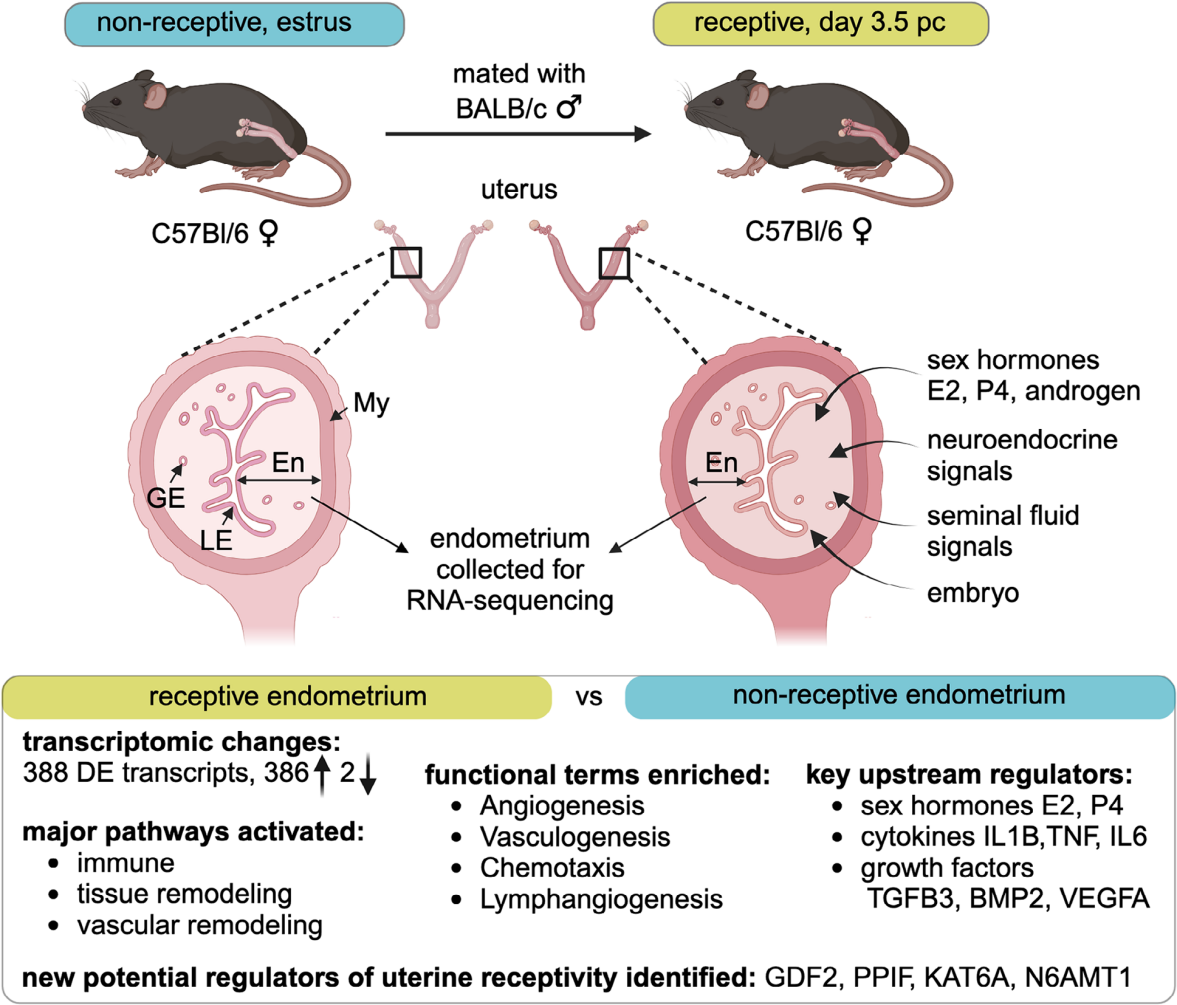
Summary of key changes identified in the day 3.5 pc endometrium compared to the estrous endometrium. Endometrium was collected from C57Bl/6 females at estrus and on day 3.5 pc after mating with BALB/c males, then RNA was extracted and sequenced. Differentially regulated genes indicated activation of immune, tissue remodeling, and vascular remodeling pathways as predicted by IPA. Enriched functional terms include Angiogenesis, Vasculogenesis, Chemotaxis, and Lymphangiogenesis. Activated upstream regulators include sex hormones, inflammatory cytokines and growth factors. Novel potential regulators for uterine receptivity are highlighted. Sex steroid hormones, seminal fluid factors, neuroendocrine signals, and embryo-derived signals all likely contribute to driving the endometrial changes. En, endometrium; GE, glandular epithelium; LE, luminal epithelium; My, myometrium; DE, differentially expressed; E2, beta-estradiol; P4, progesterone; IL1B, interleukin 1B; TNF, tumor necrosis factor; IL6, interleukin 6; TGFB3, transforming growth factor beta 3; BMP2, bone morphogenetic protein 2; VEGFA, vascular endothelial growth factor A; GDF2, growth differentiation factor 2; PPIF, peptidylprolyl isomerase F; KAT6A, lysine acetyltransferase 6 A; N6AMT1, N-6 adenine-specific DNA methyltransferase 1. Created with BioRender.com.

To allow initiation of pregnancy, the uterus must undergo major molecular and cellular changes so that receptivity for embryo attachment is acquired and implantation ensues. Implantation is the most vulnerable stage of mammalian pregnancy – for example, 50% of embryos are estimated to fail to implant in fertile women [41]. Recurrent implantation failure accounts for ~ 30% of infertility and pregnancy loss in women [42], and impaired implantation and placentation contributes to the pathophysiology of pregnancy disorders such as preeclampsia and fetal growth restriction [43, 44]. The major significance of embryo implantation underpins a substantial research effort to investigate the molecular and cellular mechanisms that generate receptivity, since these mechanisms presumably underlie the variation in receptivity between women, and within individuals from one cycle to the next. However, there are gaps in understanding how the endometrium transitions from the non-receptive, estrogen-dominated phase at ovulation, to establish a receptive state in preparation for implanting blastocysts. The current RNA-seq analysis confirms roles for several signal transduction pathways and growth factor, cytokine, and immune regulatory networks in acquisition of endometrial receptivity in mice. The data are consistent with regulation by known drivers of uterine receptivity, notably ovarian steroid hormones and seminal fluid factors. As well, the analysis reveals some new candidate upstream regulators that will inform future studies to expand understanding of implantation physiology (Fig. 6).

As expected, we found that the endometrial transcriptome is substantially different between the estrus phase and the receptive endometrium of day 3.5 pc. To enable identification of the major molecular changes accompanying endometrial transition into the receptive state, a moderate stringency logFC cut-off equivalent to FC ≥ +1.5 or ≤ -1.5) and FDR cut-off of ≤ 0.05 was used. While 80% of the 388 differentially expressed genes are protein coding genes, 20 were RIKEN genes and 54 were predicted genes with uncharacterized functions. In addition, IPA revealed that 21% of the differentially expressed transcripts encode enzymes and transcriptional regulators, consistent with substantial molecular and cellular changes taking place in the endometrium as receptivity is acquired. Notably our data show this transition is well underway prior to initiation of the decidual response, which in the mouse commences at the time the embryo first breaches the epithelium around 12–24 h later. This observation is consistent with emerging information pointing to the significance of early events in the proliferative and peri-ovulatory phase as precedents of endometrial receptivity in women [45].

The significance of sex hormone regulation of uterine receptivity and embryo implantation is well established, and our RNA-seq data align with this. IPA predicted beta-estradiol to be a top activated upstream regulator of the transcriptional changes. Prior to embryo implantation, estrogen contributes to remodeling of the uterine epithelium by limiting uterine epithelial cell proliferation and allowing differentiation [46–48]. Progesterone was also a predicted activated regulator in the day 3.5 pc endometrium. Genetic deficiency in progesterone receptors (PR) causes implantation failure in mice, due to defects in uterine epithelial cell proliferation and impaired decidualization [49]. Interestingly the data also support a role for androgens in implantation, with the androgenic sex hormone dihydrotestosterone identified as a predicted activated regulator in the receptive endometrium. This aligns with a recent report that implicates endometrial androgens in regulating decidualization [50, 51].

The RNA-seq findings point to major vascular changes and tissue remodelling events in the endometrium during acquisition of receptivity. Compared to the non-receptive endometrium, vascular functions such as Angiogenesis, Development of vasculature, Vasculogenesis, Adhesion of vascular endothelial cells, Binding of vascular cells, and Branching of vasculature were all predicted to be increased in the receptive endometrium. Angiogenesis is well known to play a critical role in healthy placental development [52]. The current results show that vascular changes occur in the endometrium even prior to embryo attachment and the decidual response, and so must initially depend on molecular signals other than paracrine factors emanating from invading trophoblasts. VEGFA and EGF were identified as upstream regulators predicted to control these vascular changes, consistent with known roles for these angiogenic factors [53–55] in uterine vascular remodelling.

Other known regulators of uterine receptivity were prominent in our analysis, adding confidence to the validity of the dataset. For instance, Indian hedgehog (*Ihh*) mRNA is substantially increased in the receptive endometrium (logFC = + 4.85). IHH expression is essential for fertility in mice through mediating progesterone actions, regulation of normal cell cycle progression, and inhibition of estrogen signaling [56, 57].

Other aspects of the dataset indicate a pro-inflammatory environment, with Chemotaxis and Lymphangiogenesis functions prominent during the peri-implantation phase [39, 58]. Immediately after conception and prior to embryo implantation, the uterus undergoes an inflammation-like response with elevated production of IL1B, IL6, and TNF [18], all of which were identified as upstream regulators in this study. These molecules are pro-inflammatory cytokines that exert immune regulatory and vasoactive effects [39]. This finding resonates with a recent report that TNF is pivotal to maternal reproductive resource allocation to balance fetal growth according to implantation site number [59]. This pro-inflammatory signaling involves recruitment into the endometrium of innate immune cells including neutrophils, macrophages, natural killer cells and dendritic cells [11, 60], which in turn regulate endometrial remodeling events that characterize transition to the receptive phase. As well as promoting maternal immune tolerance, these cells assist trophoblast invasion [61, 62], modulate uterine vascular remodeling [63, 64], and promote formation of new lymphatic vessels at the maternal-fetal interface [65].

Several of the signaling factors predicted to drive these immune-regulatory pathways are present in semen or induced in the endometrium by seminal fluid contact. Seminal fluid factors include transforming growth factor beta (TGFB) and TLR4 ligands present in seminal plasma [66], and cytokines TNF and IL6 induced in endometrial epithelial cells after seminal fluid stimulation [67]. Their prominence in this dataset is consistent with extensive evidence linking seminal fluid with regulation of immune adaptation to pregnancy. The cellular changes in the female tract initiated by seminal fluid contact provoke generation of immune suppressive Treg cells and their recruitment from local lymph nodes, to constrain inflammation and support maternal immune tolerance [18].

Of the seminal fluid signals implicated in acquisition of endometrial receptivity, TGFB is most critical for promoting Treg cells [16, 68, 15, 69]. The TGFB3 isoform was predicted to be activated during the pre-implantation phase with an activation Z-score of + 2.4 (Fig. 4B). In mice and humans, TGFB3 is considered a major seminal plasma signaling mediator that together with TGFB1 and TGFB2 induces endometrial cytokines CSF2, IL1A and IL6 [16, 67]. Interestingly, the data point to a possible role of AGT in seminal fluid [70, 71] in regulating endometrial changes preceding implantation.

This study supports our previous findings that TLR4 signaling is activated in the uterus in the peri-conception period [18, 67]. TLR4 signaling pathway target NFκB and adaptor protein MYD88 are predicted to drive gene expression changes in the early receptive endometrium. This finding is consistent with previous reports that the TLR4 signaling pathway is activated by seminal fluid at conception [67]. TLR4 activation contributes to Treg cell generation and recruitment and is crucial for optimal embryo implantation [18, 67]. Several TLR4 ligands are present in semen such as beta-defensins, heat shock proteins, and S100 proteins [66], but their specific signaling actions in the female tract are not yet defined. Analysis of effects of males deficient in specific seminal fluid components will be necessary to delineate the relative contribution of the male and female to the origin of these upstream regulators.

A systematic literature search revealed potential upstream regulators not previously identified as involved in uterine receptivity or embryo implantation. One of these, GDF2 is a member of the BMP family that is implicated in angiogenesis regulation via interaction with endoglin [72, 73], a key molecule involved in receptivity [74]. Roles for GDF2 in vascular maturity and remodeling [75], and lymphatic vascular network development [76], are reported. GDF2 may also contribute to leukocyte recruitment into the uterus through inducing TLR4 expression in endothelial cells [77]. Thus GDF2 is a strong candidate for facilitating uterine receptivity through regulating vascular and immune changes.

Additional novel molecules not previously linked with endometrial receptivity include KAT6A, N6AMT1, and PPIF. KAT6A is a member of the acetyltransferase family and an oncogene in multiple cancers [78, 79]. It is associated with acetylation of TGFB signaling mediator SMAD3 [80], and so may facilitate TGFB-mediated effects on immune tolerance. N6AMT1 is a DNA methyltransferase with limited information on its functions in normal physiology. The predicted activation of KAT6A and N6AMT1 in the early receptive endometrium is consistent with epigenetic modification playing a role in uterine receptivity [81, 82]. Lastly, PPIF is a mitochondrial protein that mediates mitochondrial permeability and its deficiency leads to improved mitochondrial functions in adult mice [83]. Since mitochondrial function is required in antigen-specific T cell activation [84], the predicted inhibition of PPIF may facilitate uterine receptivity by enhancing the maternal T cell response and contributing to generation of immune tolerance. Given the potential relevance of these molecules to important aspects of embryo implantation, further investigation to define their specific roles is warranted.

A strength of the current study is that several analytical tools were utilized to generate a complete understanding of the sequencing data. As well as IPA software, public domain analytical tools including GO, KEGG pathway analysis and Reactome analysis were utilized. These analyses also indicate substantial molecular and cellular changes in the uterus prior to embryo implantation. All four platforms highlighted that enriched functional terms associated with vascular and tissue remodelling are prominent. There were minor differences in terms identified by different platforms. For instance, immune pathways were only identified by IPA, while ECM-associated terms were only enriched using GO, KEGG and Reactome, which likely reflects differences in gene annotation associated with the different tools.

A limitation of this study is that many gene expression changes in minor cell components are unlikely to be detectable. Endometrial stromal cells or epithelial cells comprise at least two thirds of the cells in the endometrium at both time points examined, and so would account for most of the transcripts identified. Given decidualization does not occur until after implantation, the cellular composition of both sample types is likely comparable. While immune cells make up nearly one third of cells in the endometrial stroma, they are comprised of a range of distinct subpopulations with different transcriptional profiles, so that most subsets are minor populations [26]. Given we used stringent cutoff criteria to define differentially expressed genes, it is unsurprising that the majority of the genes identified are related to cellular processes other than immune functions. Single cell sequencing approaches will be better suited to investigating how immune cells contribute to endometrial receptivity.

## Conclusions

In summary, these findings confirm that major transcriptional changes take place in the endometrium of the mouse after ovulation and prior to embryo implantation. Our findings are consistent with the literature, and consolidate extensive data showing adaptations involving the maternal immune and vascular systems. The data demonstrate that these changes commence prior to blastocyst attachment and trophoblast invasion, and the accompanying decidual transformation. Our results advance understanding of the precise molecular mechanisms that govern uterine receptivity, by identifying genes not previously associated with receptivity acquisition. In particular, GDF2, KAT6A, N6AMT1 and PPIF may be relevant to the immune changes required for receptivity to embryo implantation and warrant investigation in future studies to define their physiological significance in the mouse, and possible clinical significance in women.

## Methods

### Animals and tissue collection

All the animal experiments performed were approved by the University of Adelaide Animal Ethics Committee (approval number M/2014/023). Mice were provided food and water *ad libitum* and housed under controlled temperature and a 12 L/12D cycle in a pathogen-free facilities of Laboratory Animal Services (LAS) at the University of Adelaide, Australia. C57Bl/6 females and BALB/c males were purchased from the University of Adelaide Central Animal Facility.

C57Bl/6 females at 8–14 weeks were housed with BALB/c males and checked each morning between 0900 and 1100 h for copulatory plugs. Mice with a copulatory plug were designated as day 0.5 pc and were separated from males and housed in groups of 1–3 until they were killed by cervical dislocation at 1200 h on day 3.5 pc. The uterus was excised and placed in cold RNase-free PBS, then fat, mesentery and blood vessels were removed with the aid of a dissection microscope (Olympus, New York, NY, USA). Uteri were then transferred into fresh RNase-free PBS and slit open lengthwise to expose the endometrium. Endometrial tissue was scraped from the myometrium using a sterile razor blade (ProSciTech, Queensland, Australia) into 700 ${\mu }$l of Trizol lysis reagent (Thermo Fisher Scientific, Waltham, MA), and the myometrium was discarded. Endometrium was then homogenized with 0.6 g of 1.5 mm ceramic beads using the Powerlyzer® 24 Bench top bead-based homogenizer (MoBio Laboratories, Carlsbad, CA) at a setting of one cycle at 3500 rpm for 10 s, snap-frozen in liquid nitrogen, and stored at -80°C. Endometrial tissue was also collected from virgin C57Bl/6 females at 2200–2300 h (around the time of ovulation) on the day of detection of proestrus based on vaginal cell morphology [85].

### Isolation of total RNA

Total RNA was extracted into Trizol (Thermo Fisher Scientific) and DNase-treated with TURBO DNA-free™ (Life Technologies) according to the manufacturer’s instructions. DNA removal was confirmed by quantitative-polymerase chain reaction (qPCR) using genomic DNA (gDNA)-specific primers (0.25 µm; 5’ primer - TGTGATGGTGGGTATGGGTC, 3’ primer - ACACGCAGCTCATTGTA) as described [86]. RNA concentration was measured using a Nano-drop Spectrophotometer ND-1000 (Thermo Fisher Scientific), and RNA integrity was assessed by RNA Agilent Bioanalyzer (Agilent Technologies, Santa Clara, CA) to ensure all RNA samples had an RNA integrity number of > 7.

### RNA-sequencing, bioinformatics and statistics

For each RNA sample, 700 ng of total RNA in a volume of 50 𝜇l was prepared and sent to the Centre for Brain Genomics at the University of Queensland for RNA-seq. Samples underwent library prep using TruSeq Stranded total RNA Library Prep Kit (Illumina, San Diego, CA, USA), followed by paired-end next-generation sequencing on the Illumina HiSeq2000 platform to a depth of 30 million reads per sample. All sequencing FASTQ files were processed with a standard RNA-seq pipeline. Sequences were assessed for quality using *FastQC*, sequencing adapters trimmed using *AdapterRemoval* version 2 and aligned to the GRCm38/mm10 mouse reference genome sequence (https://www.ncbi.nlm.nih.gov/assembly/GCF_000001635.20/) using *STAR* [87]. Gene counts were identified using *featureCounts* [88], which were then transferred to R for downstream analysis. All downstream differential expression analyses were carried out using R/Bioconductor packages *limma-voom* [89] and *edgeR* [90, 91]. The criteria for designation of differentially regulated genes was false discovery rate adjusted-p value (FDR) ≤ 0.05 with log_2_ fold-change (logFC) ≥ + 0.583 or ≤ -0.583 (equivalent of FC ≥ + 1.5 or ≤ -1.5). To investigate gene pathways and upstream regulators influenced during the peri-implantation period of pregnancy, differentially expressed genes were analysed using Ingenuity Pathway Analysis (IPA) software version 65367011 (Ingenuity Systems, Redwood City, CA). Additional functional enrichment analysis was carried out using Gene Ontology (GO), Kyoto Encyclopedia of Genes and Genomes (KEGG)(with permission from Kanehisa Laboratories, Japan) [92], and Reactome. The RNA-seq dataset discussed in this manuscript has been deposited in the National Center for Biotechnology Information’s Gene Expression Omnibus GSE223136 [93]. The two groups of endometrial tissue samples reported in this study (estrus and day 3.5 pc) are part of a dataset that includes five experimental groups in total. RNA-sequencing and data normalization were performed on all groups simultaneously and data from the other three sample groups will be reported separately. The work flow and code for the analysis are provided at the following Github link [94].

### Electronic supplementary material

Below is the link to the electronic supplementary material.

Supplementary Material 1

Supplementary Material 2

Supplementary Material 3

Supplementary Material 4

Supplementary Material 5

## Data Availability

The data supporting the findings and conclusions of this research article are available at the National Center for Biotechnology Information’s Gene Expression Omnibus GSE223136 and can be accessed at https://www.ncbi.nlm.nih.gov/geo/query/acc.cgi?acc=GSE223136.
